# Simplified Evaluation of Shear Stiffness Degradation of Diagonally Cracked Reinforced Concrete Beams

**DOI:** 10.3390/ma16134752

**Published:** 2023-06-30

**Authors:** Kaiqi Zheng, Siyuan Zhou, Yaohui Zhang, Yang Wei, Jiaqing Wang, Yuxi Wang, Xiaochuan Qin

**Affiliations:** 1 College of Civil Engineering, Nanjing Forestry University, Nanjing 210037, China; k.zheng@njfu.edu.cn (K.Z.); 8210610638@njfu.edu.cn (S.Z.); jiaqingw@njfu.edu.cn (J.W.); wyxwyx11@126.com (Y.W.); 2 State Key Laboratory of Mechanical Behavior and System Safety of Traffic Engineering Structures, Shijiazhuang Tiedao University, Shijiazhuang 050043, China; 3 State Key Laboratory of High Performance Civil Engineering Materials, Jiangsu Research Institute of Building Science, Nanjing 210008, China; qinxiaochuan@cnjsjk.cn

**Keywords:** reinforced concrete beam, diagonal cracking, shear deformation, shear stiffness, strut angle, tension stiffening, stiffness degradation

## Abstract

Shear cracking in concrete box-girder bridges, which could cause excessive deflection during the serviceability limit state, cannot be effectively avoided by code-guided design. While elastic shear deformation only accounts for a small proportion of total deformation for un-cracked reinforced concrete (RC) beams, the magnitude of after-cracking shear deformation becomes comparable to flexural deformation for RC beams. However, there is still a lack of practical models to predict the after-cracking shear deformation of RC beams. First, six thin-webbed I beams were tested to investigate the shear stiffness degradation mechanism and the decrease ratio. Then, a very simple truss strut angle formula, which is the crucial parameter for shear stiffness, was established. Furthermore, a stiffness degradation rule for partially cracked beams was proposed considering the influence of concrete tension stiffening, which is essential for predicting the development process of after-cracking shear deformation. Finally, directly measured shear strains were used to validate the proposed shear stiffness model. The results showed that the shear stiffness drops to about 30~40% of the original stiffness after the first diagonal crack, and the remaining shear stiffness is only about 10% of the original one when the stirrup yields. Increasing the stirrup ratio is a more effective method to control shear stiffness degradation for diagonally cracked RC beams. Also, the proposed shear stiffness model well captures the main features of the shear stiffness degradation, and it provides a relatively accurate prediction of the equivalent shear stiffness at the post-cracking stage.

## 1. Introduction

As is well known, the deformation of concrete beams mainly consists of two parts: bending deformation and shear deformation [1]. Generally, the deformation of beams is mainly bending deformation, and the magnitude of shear deformation is small, which can be ignored [2,3]. However, for long-span concrete box-girder bridges commonly used in bridge engineering, this assumption may cause deviations [4]. Especially for the thin-webbed box girder with diagonal cracking, its shear deformation may be equivalent to the bending deformation [5].

According to a finite element analysis of a thin-webbed box-girder bridge with large web height [4], the magnitude of elastic shear deformation under load is considerably large and may reach a level that cannot be ignored. Scholars’ experiments on thin-webbed concrete beams [1,6] have shown that: after the appearance of diagonal cracks, the shear deformation of the beam will significantly increase, with a shear deformation ratio of over 30% of the total deformation. Further, according a survey on an existing concrete box-girder bridge conducted by the Research Institute of Highway Ministry of Transport of China [7], diagonal web cracking occurs in more than 90% of this type of bridge. This indicates that with the development of diagonal cracks in the web, the impact of shear deformation on the deflection of thin-webbed beam bridges will be greater and should be taken seriously. Considering that the influence mechanism of diagonal cracks on deformation is extremely complex, conducting a shear test on thin-webbed concrete beams is the most effective way to discover and summarize its laws of influence [8,9].

So far, although thousands of shear tests have been conducted on concrete beams, the vast majority of specimens have beam heights not exceeding 500 mm, and only a few beams without web reinforcement have reached heights exceeding 1000 mm [10,11,12,13,14,15,16,17,18,19,20]. Due to the influence of size effects, these specimens cannot truly reflect the actual stress behavior of large-sized beams widely used in practical engineering. And, most of the existing experiments have focused on shear strength and failure mode, with less attention paid to the contribution of shear deformation after shear cracking.

As RC beams are subjected to the combined action of the shear and bending moment, the corresponding shear and flexural deformation are coupled with each other. It is difficult to measure the shear deformation separately. Experimental research on post-crack deformation of RC beams is also limited to the comparison of total deformation [21,22,23]. Although there are a few quantitative experimental studies focusing on direct shear deformation of concrete beams after shear cracking, in which a decoupling technique of shear deformation and bending deformation has been proposed [24,25], the data are limited and are not compatible with variable depth beams. Based on the iso-parametric concept from the finite element method, a theoretical deformation decoupling method for variable depth beams was proposed [26], which provides a new approach for the direct measurement of shear deformation.

In another aspect, researchers proposed calculating methods for after-cracking shear deformation. Based on the assumption of homogeneous characteristics for cracked concrete, the modified compression field theory (MCFT) could provide the whole load-deformation curve for RC beams [27,28,29,30]. However, iterative calculations are required, which are not suitable for the rapid evaluation of existing bridges. The variable angle truss model (VATM)-based calculating theory for shear deformation prediction is proposed by Pan et al. [7], whereby constant tangent stiffness degradation rules are suggested for diagonally cracked RC beams, which is very simple and conservative for engineering practice. However, efforts are still needed to achieve maximum optimization in computational accuracy and simplicity [31,32].

This study aims to obtain the shear stiffness degradation law of large-scale thin-webbed concrete beams and to propose a simplified and practical prediction method, which can be further applied to concrete box-girder bridges with diagonal web cracks. The degradation of after-cracking shear stiffness is studied by experimental tests. Further, a simplified and practical model is proposed to depict the degradation of shear stiffness. And finally, shear deformation test data in this paper and in the literature are used to verify the accuracy and applicability of the proposed simplified shear stiffness degradation model.

## 2. Experimental Test of Shear Stiffness Degradation

### 2.1. Test Object and Design Concept

To study the impact of diagonal cracks on the degradation of shear stiffness and development of shear deformation of concrete thin web beams, a direct shear measurement test was conducted on six concrete thin-webbed constrained beams. The main experimental objectives include:

(1) Achieve continuous direct measurement of shear deformation before and after diagonal cracks in the concrete web;
(2) Analyze the amplitude of changes in shear deformation values before and after shear cracking and study the degree of influence of diagonal cracks on shear deformation;
(3) Study the degradation law of shear stiffness after the development of diagonal cracks.

In order to achieve the above experimental objectives, the main experimental ideas used include:

(1) Using large-scale thin-webbed I-shaped cross-section specimens to better simulate the stress behavior of thin web bridges, while facilitating the testing of web strain and the observation of diagonal cracks.
(2) Adopting a reinforcement design with “strong bending and weak shear“ concept, ensuring the priority occurrence and full development of diagonal cracks, with a focus on observing the impact of diagonal cracks on shear deformation and shear stiffness.
(3) Constrained beams are used to investigate the shear performance of concrete beams under different combinations of bending and shear internal forces.
(4) The effects of inclined bottom chord on diagonal crack and shear strength were investigated by using two types of specimens, namely, equal-height beam and variable-height beam.
(5) Propose a strain-based shear deformation calculation method for arbitrary quadrilateral lattices, achieving direct peeling measurement of bending deformation and shear deformation.

### 2.2. Specimen Parameters and Setup

**Figure 1:**
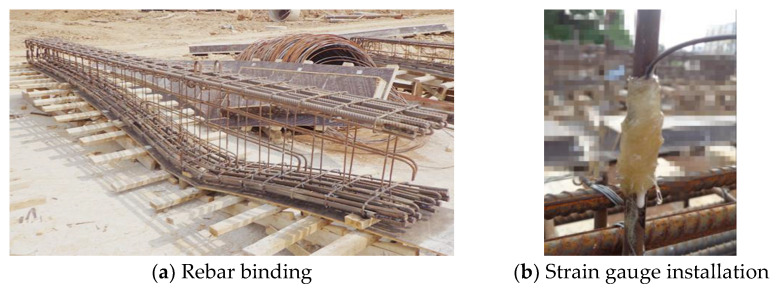

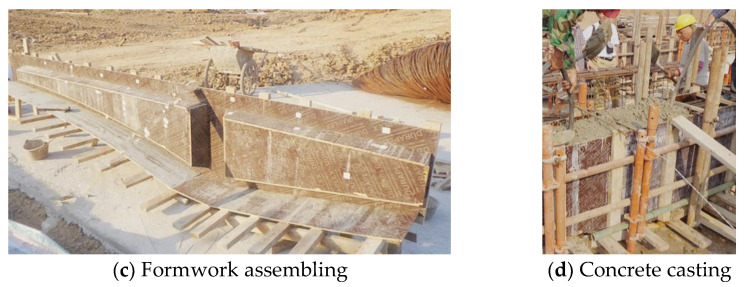
Fabrication process of specimens.

A total of 6 I-shaped cross-section specimens were made and divided into two groups: Group BC consisted of 2 beams of equal height; Group BV consisted of 4 variable-height beams, with the upper flange of the cross-section horizontal and the lower flange height varying in a parabolic manner. All the specimens were constructed in two batches on site (Figure 1). The width of the beam web is 100 mm, and the beam length is 5400 mm.

**Figure 2:**
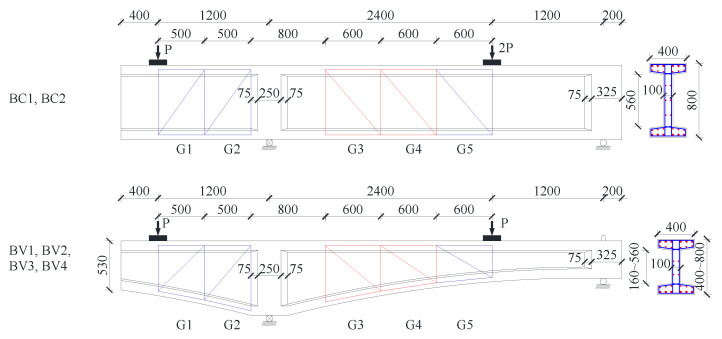
Beam dimension and strain measuring lattice arrangement (unit: mm).

**Figure 3:**
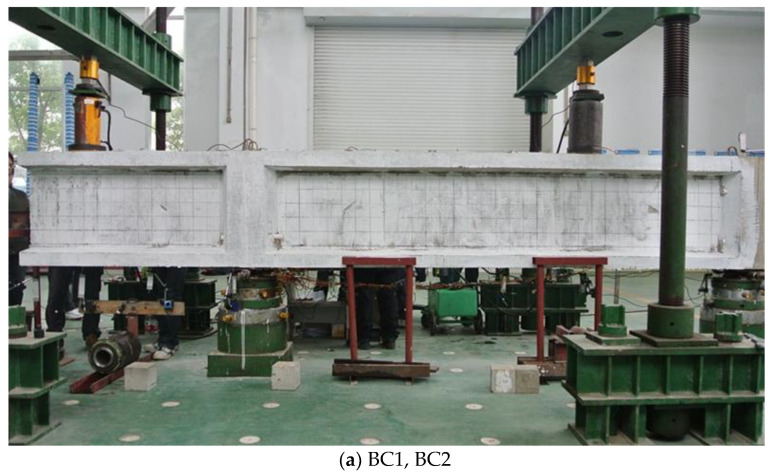

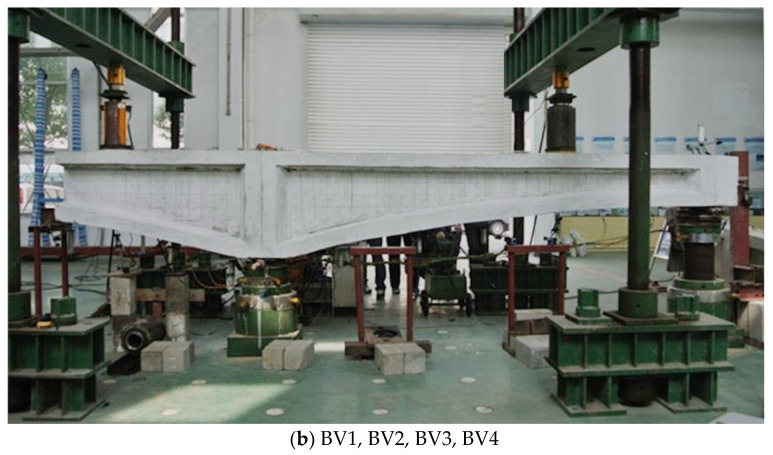
Test setup.

All the specimens are simply supported with a cantilever, which were loaded with two concentrated loads at the cantilever end and within the simply supported span, respectively. The ratio of cantilever load to span load is 1:2 for BC specimens, and 1:1 for BV specimens. The detailed dimensions and elevation layout of the component are shown in Figure 2 and Figure 3, The detailed arrangement of the measuring lattice and corresponding calculation method could refer to a pre-publication focusing on the measuring technology [26].

For the specimens, D25 bars and D12 bars with a total cross section area of 3624 mm^2^ and average yield strength of 497 MPa were used as longitudinal bars, which were arranged symmetrically in top and bottom flanges. D8 round bars (two legs) with average yield strength of 326 MPa were arranged as stirrups at a spacing of 200 mm (*ρ*_v_ = 0.5%) or 250 mm (*ρ*_v_ = 0.4%) for deferent beams along the whole span. The average concrete cylinder strengths were 39.0 MPa and 36.0 MPa for batch I (BC1, BV1, BV2) and batch II (BC1, BV1, BV2), respectively. It must be mentioned that excessive longitudinal reinforcement is arranged to prevent early flexural failure. The specimen number and main design parameters are shown in Table 1, with the changing parameters being concrete strength and reinforcement ratio.

### 2.3. Specimen Failure Modes

**Figure 4:**
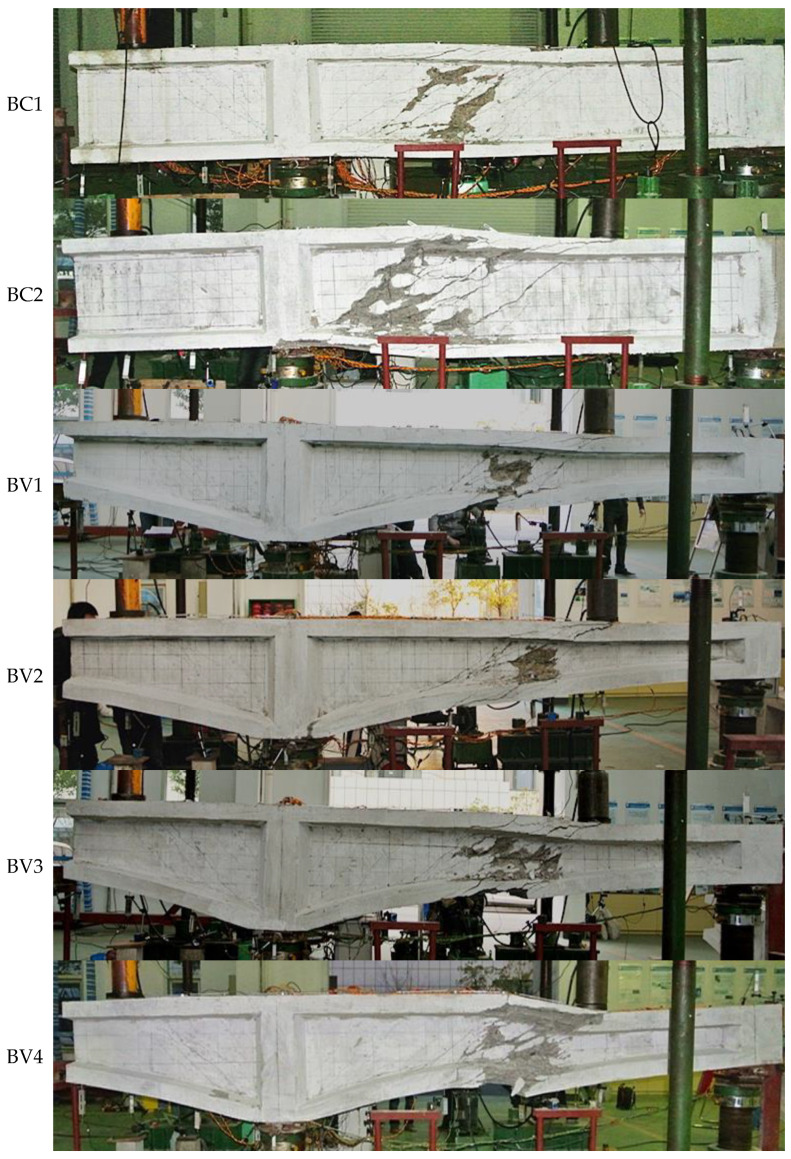
Failure mode of specimens.

The final failure mode of all 6 specimens is shear failure, manifested as the yielding of web stirrups and concrete crushing at the bending reverse point, as shown in Figure 4.

The test reveals that the development of bending cracks was slow or almost non-existent during the loading process, and the strain increment of longitudinal reinforcement was small and did not reached yield [10]. With the appearance of diagonal cracks, their development is relatively rapid, quickly developing from the middle of the web to the upper and lower flanges, and gradually penetrating the entire web. With the formation of the main diagonal crack, the stirrups at the diagonal crack also yielded quickly. Afterwards, the number, width and range of diagonal cracks further expanded, and the stirrups of different shear span also yielded one after another. Finally, the concrete of the beam web at the bending reverse point collapsed, and the deformation increased sharply, declaring the failure of the specimen.

Though the first diagonal crack occurs at the cantilever span with maximum shear forces, all specimens failed in shear uniformly at the bending reverse point The positive and negative bending moment on both sides of the bending reverse point intensifies the shear deformation of the concrete web, ultimately leading to the tearing of the concrete web and the diagonal compression of the concrete web. The phenomenon reflects that the bending reverse point section is the weakest position for shear failure of continuous beams (constrained beams).

### 2.4. Observed Shear Stiffness Degradation

Besides failure modes, the sequence of shear crack, strain development and yielding of stirrup, and failure load of each shear span can refer to an earlier publication [26]. Here, we only focus on the degradation of shear stiffness. Shear stiffness is the most important measure of structural shear deformation. In the elastic stress stage, the shear stiffness *K*_e_ of the section can be expressed as *GA*_v_, and considering the Poisson’s ratio, it becomes *EA*_v_/[2(1 + *μ*)]. After cracking, due to the destruction of structural continuity, the shear stiffness is no longer equal to elastic shear stiffness. Generally, the nominal shear stress of the test beam after cracking can be taken as *τ*, which is the ratio of shear force *P* to the shear cross-sectional area *A*_v_. Based on the stress–strain relationship, the equivalent shear stiffness *K*_eq_ of the shear cracked specimen is defined by the following equation,

(1) $$K_{\mathrm{eq}}=\frac{\tau }{\bar{\gamma }}A_{v}=\frac{P}{\bar{\gamma }}$$

To study the variation of shear stiffness after the development of diagonal cracks, a shear stiffness degradation factor is defined as *λ*, namely, the ratio of equivalent shear stiffness *K*_eq_ to elastic shear stiffness *K*_e_,

(2) $$\lambda =K_{\mathrm{eq}}/K_{e}$$

**Figure 5:**
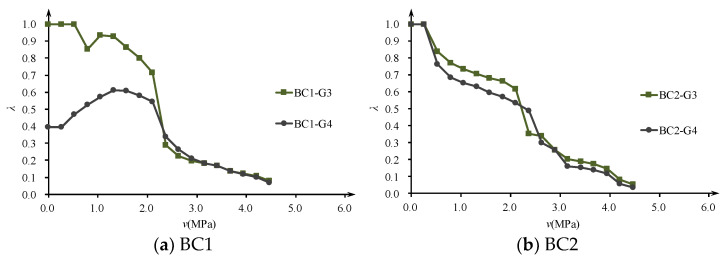

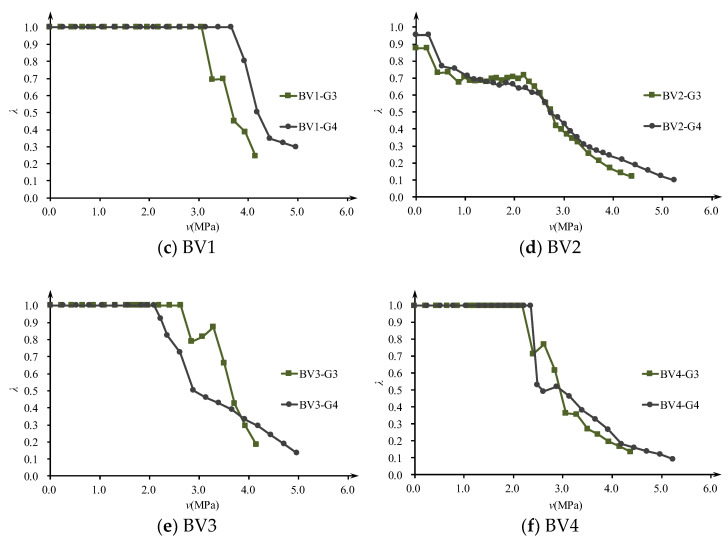
Degradation law of shear stiffness of specimens.

Considering that the tested shear strain in the elastic stage is very small, if the calculated *λ* > 1, take *λ* = 1. The shear stiffness reduction factor *λ* of each observation lattice can be plotted as a function of nominal shear stress *τ*, as shown in Figure 5. It can be seen that except for specimen BV1, the shear stress levels of all specimen frame G3 and G4 lattices with significantly reduced shear stiffness are around 2MPa, which is in good agreement with the initial shear crack load. This indicates that the shear stiffness reduction factor *λ* can properly reflect the impact of diagonal web cracks on shear stiffness.

Comparing the degradation curves of shear stiffness of specimens with different concrete strength grades (BV1 and BV3, BV2 and BV4) and different reinforcement ratios (BV1 and BV2, BV3 and BV4), it was found that increasing the concrete strength grade (mainly the elastic modulus) and increasing the reinforcement ratio can both improve the shear stiffness after cracking. The contribution of increasing the reinforcement ratio to the remaining shear stiffness is more significant after shear cracking, and the suppression of shear deformation is more effective. Generally, after the diagonal cracking of the specimen, the shear stiffness is about 30~40% of the original, but when the stirrup yields, the remaining shear stiffness is only about 10%.

## 3. Proposed Shear Stiffness Degradation Model

### 3.1. Fully Diagonally Cracked Shear Stiffness

To evaluate the cracked shear stiffness, the truss model is recommended by scholars [33,34,35,36]. For slender beams, the inclined cracks are roughly parallel to each other. Therefore, a standard VATM can be used for analysis. As the truss model ignores the tensile stresses between cracked concrete, it is only suitable for calculating the shear stiffness of fully diagonally cracked RC beams. If the strut angle *θ_u_* is determined, the corresponding shear stiffness *K_u_* can be expressed as,

(3) $$K_{u}=\frac{n\rho_{v}\cot^{2}\theta_{u}}{1+n\rho_{v}\csc^{4}\theta_{u}}E_{c}A_{v}$$

where *K_u_* is the fully diagonally cracked shear stiffness, *n* is the ratio of *E_s_* to *E_c_*, *E_s_* is the modulus of elasticity of reinforcing steel, *ρ_v_* is the stirrup ratio, and *θ_u_* is the strut angle.

### 3.2. Ultimate Shear Stiffness Degradation Factor

Similarly to Equation (2), we can define ultimate shear stiffness degradation factor *λ_u_*, which is equal to the ratio of *K_u_* and *K_e_*,

(4) $$\lambda_{u}=\frac{K_{u}}{K_{e}}=\frac{2n(1+\mu )\rho_{v}\cot^{2}\theta_{u}}{1+n\rho_{v}\csc^{4}\theta_{u}}$$

The factor *λ_u_* is defined as the shear stiffness degradation factor, which reflects the decreasing magnitude of shear stiffness at the fully cracked stage. As shown in Equation (4), the main parameters that influence *λ_u_* are the stirrup ratio *ρ_v_* and the strut angle *θ_u_*. If the only unknown parameter *θ_u_* is determined, *λ_u_* can be easily calculated by Equation (4).

### 3.3. Determination of Strut Angle θ_u_

Scholars have already proposed various solving methods for the strut angle [33,34,35], most of which employed the minimum energy principle or plasticity theory. Their findings imply that the strut angle closely relates to the stirrup ratio, longitudinal reinforcement ratio, or concrete strength.

For calculating the shear stiffness calculation of fully diagonally cracked slender RC beams, Pan et al. [8] suggested that the strut angle can be calculated by Equation (5). It accounts for the influence of the web and longitudinal reinforcement.

(5) $$\theta_{u}=\arctan\left[{\left(\frac{1+\frac{1}{n\rho_{s}}}{1+\frac{1}{n\rho_{v}}}\right)}^{0.25}\right]$$

In addition, He et al. [9] derived the strut angle of slender beams based on the lower-bound theorem of plasticity (Equation (6)). It accounts for the influence of the stirrup ratio and the concrete strength while assuming that the longitudinal reinforcement will not yield before shear failure. The key parameter for the equation *ω* is the mechanical web reinforcement ratio, and *ω* = *ρ_v_f_yv_*/*f’_c_*, *f’_c_* is the compressive strength of concrete.

(6) $$\theta_{u}=\arcsin\left(\sqrt{0.23\left(\omega +\sqrt{\omega^{2}+13\omega }\right)}\right)$$

**Figure 6:**
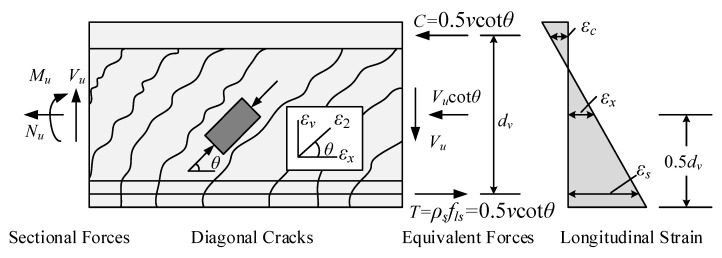
CFT adopted for shear analysis of RC beam.

For simplification, compression field theory [27] is adopted for beam shear analysis to determine the strut angle (see Figure 6). The formulation process assumes that concrete beams are subject to service load, under which the steel bars and the inclined concrete struts behave linearly elastically.

The equilibrium of average stress and compatibility of average strain in a beam section are summarized in Equations (7)–(13), in which the longitudinal strain *ε_x_* at the middle height is assumed to be 0.5 times the longitudinal strain *ε_s_* at the center of longitudinal steel bars [3].

(7) $$\rho_{v}f_{v}=v\;\tan\theta_{u}$$

(8) $$\rho_{s}f_{ls}=0.5v\cot\theta_{u}$$

(9) $$f_{2}=v\left(\tan\theta_{u}+\cot\theta_{u}\right)$$

(10) $$\tan^{2}\theta_{u}=\frac{\varepsilon_{x}+\varepsilon_{2}}{\varepsilon_{z}+\varepsilon_{2}}$$

(11) $$\varepsilon_{2}=\frac{f_{2}}{E_{c}}$$

(12) $$\varepsilon_{x}=\frac{\varepsilon_{s}}{2}=\frac{f_{ls}}{2E_{s}}=\frac{f_{ls}}{2nE_{c}}$$

(13) $$\varepsilon_{z}=\frac{f_{v}}{E_{s}}=\frac{f_{v}}{nE_{c}}$$

where *v*, *f_v_* and *f_ls_* are the shear stress, stirrup stress and longitudinal reinforcement stress, respectively, and *ε_x_*, *ε_s_*, *ε_z_* and *ε*_2_ are the longitudinal strain, longitudinal strain at the center of longitudinal steel bars, vertical strain and main compression strain, respectively.

From Simultaneous Equations (7)–(13), we can obtain,

(14) $$\theta_{u}=\arctan\left[{\left(\frac{1+\frac{1}{4n\rho_{s}}}{1+\frac{1}{n\rho_{v}}}\right)}^{0.25}\right]$$

Equation (14) is mainly influenced by the web and longitudinal reinforcement ratio.

### 3.4. Comparison of θ_u_ with Other Methods

To investigate the validity of angle prediction for slender beams, parameter analysis and comparison are performed according to Pan et al. [8], He et al. [9] and the proposed Equation (14). Parameter values of the reference specimen are *ρ_v_* = 0.5%, *ρ_s_* = 2%, *f’_c_* = 50MPa and *f_yv_* = 400MPa. Figure 6 shows how the strut angle changes as a function of stirrup ratio *ρ_v_*, longitudinal reinforcement ratio *ρ_s_* and concrete strength *f’_c_*, respectively.

**Figure 7:**
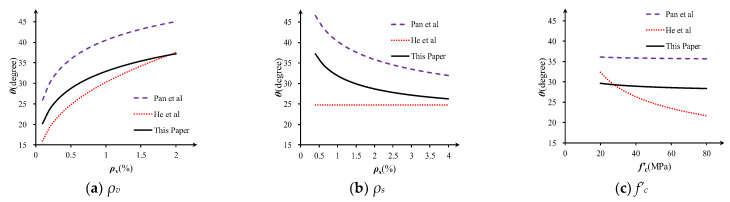
Comparison of different calculation methods of strut angle *θ* [8,9].

As is shown in Figure 7, the proposed equation gives an intermediate prediction of strut angle *θ_u_*, while *θ*_Pan_ predicts the highest value, and *θ*_He_ predicts the lowest. All three angles are affected by stirrup ratio *ρ_v_*, along with which the predicted angle grows (Figure 7a). Meanwhile, both *θ_u_* and *θ*_Pan_ reflect the inverse relationship between longitudinal reinforcement ratio *ρ_s_* and the strut angle (Figure 7b). *θ*_He_ ignores the influence of longitudinal reinforcement ratio *ρ_s_* but emphasizes the importance of concrete strength *f’_c_* (Figure 7c), and it may cause larger deviations for specimens with low longitudinal reinforcement ratio or high concrete strength.

### 3.5. Proposed Degradation Rules

In spite of the fact that we have obtained the elastic shear stiffness and the fully cracked shear stiffness, it is still difficult to evaluate the effective shear stiffness *K_eff_* of a partially diagonal cracked RC beam. As the transition from elastic stiffness to post-cracking stiffness is very complicated and is controlled by many parameters, establishing an exact and quantified expression for *K_eff_* is almost impossible.

**Figure 8:**
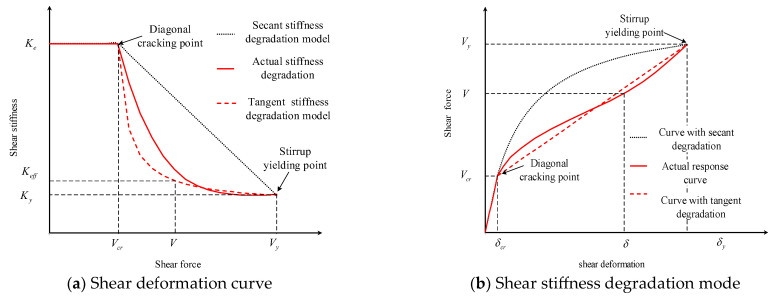
The shear stiffness degradation of diagonally cracked RC beam.

As is shown in Figure 8, the simplest and most ideal shear stiffness degradation model is the secant stiffness linear degradation model, which assumes that the post-cracking secant shear stiffness will degrade linearly with the shear force (Figure 8a), and it tends to give an unsafe prediction of shear deformation under service state. However, another shear stiffness model, namely, the constant tangent stiffness degradation model, which assumes that the post-cracking tangent shear stiffness will keep constant before stirrup yielding (Figure 8b), is only suitable for thin-webbed beams and tends to give a larger shear deformation prediction.

For RC beams, tension stiffening arises from tension carried by the concrete between the cracks (whether flexural or shear cracks). This contribution decreases with an increasing load after the member has cracked. To simulate the tension stiffening effect, the effective moment of inertia *I_e_* approach introduced by Branson [37] facilitates a gradual transition from un-cracked to a fully cracked section as the ratio of service load moment *M_a_* to cracking moment *M_cr_* increases.

The ACI 318 standard [2] adopts Branson’s degradation method when calculating the bending stiffness after flexural cracking. To make the deformation calculation equation uniform and simple, we recommend using a degradation criterion similar to Branson to reflect the effect of tensile hardening. The recommended shear stiffness degradation formula is as follows,

(15) $$K_{eff}=K_{u}+{\left(\frac{V_{u}-V}{V_{u}-V_{cr}}\right)}^{3}\left(K_{e}-K_{u}\right)\leq K_{e}$$

where the diagonal cracking load *V_cr_* and stirrup yielding load *V_u_* can be calculated by Equations (16) and (17), respectively.

(16) $$V_{cr}=0.17\sqrt{f_{c}^{\prime }}b_{w}d$$

(17) $$V_{u}=V_{cr}+\rho_{v}f_{yv}b_{w}d_{v}\cot\theta_{u}$$

## 4. Test Verification and Discussion

### 4.1. Experiment Introduction

The shear deformation of 12 beam lattices in 6 thin web-restrained beams was directly measured and analyzed with self-designed strain-measuring lattices. Further, the shear deformation test of six beams conducted by Hansapinyo [24] was used to verify the proposed degradation model. Data for a total of 16 shear deformation measurement lattices are used for experimental verification. The main parameters of the specimens are listed in Table 1. Hansapinyo’s test gives detailed shear strain test results, in which electronic transducers were also used to measure the normal and shear strains of each lattice based on the rosette concept.

As the stress distribution is disturbed by local point load in D regions (discontinuity regions, such as the lattice regions of G1, G2 and G5 shown in Figure 2), the direct strut component joins in the force transfer mechanisms in addition to the flexural and shear components. Consequently, the total deformation consists of not only flexural deformation and shear deformation but also the deformation of direct strut compression. Mean shear strain in these D regions becomes insignificant. Therefore, shear strain analyses are only performed on the lattice where the local point load disturbances are negligible (Lattice G3 and G4).

### 4.2. Comparing Results and Discussion

**Figure 9:**
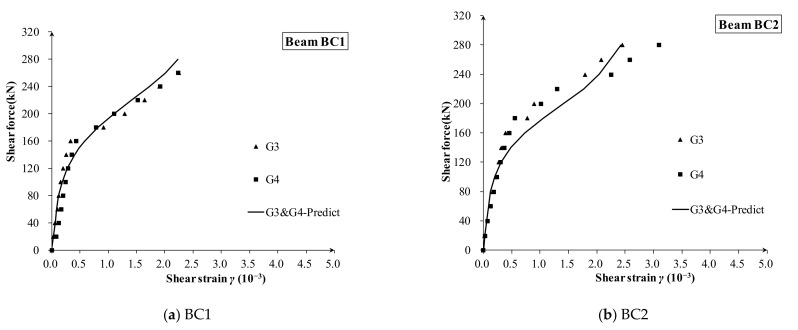
The shear strain of each measuring lattice in specimens BC1 and BC2.

**Figure 10:**
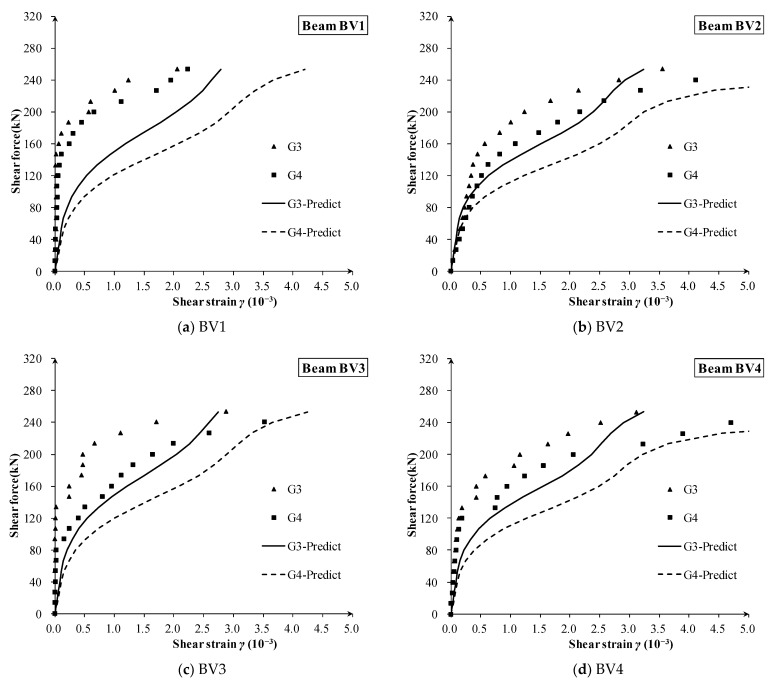
The shear strain of each measuring lattice in specimens BV1, BV2, BV3 and BV4.

**Figure 11:**
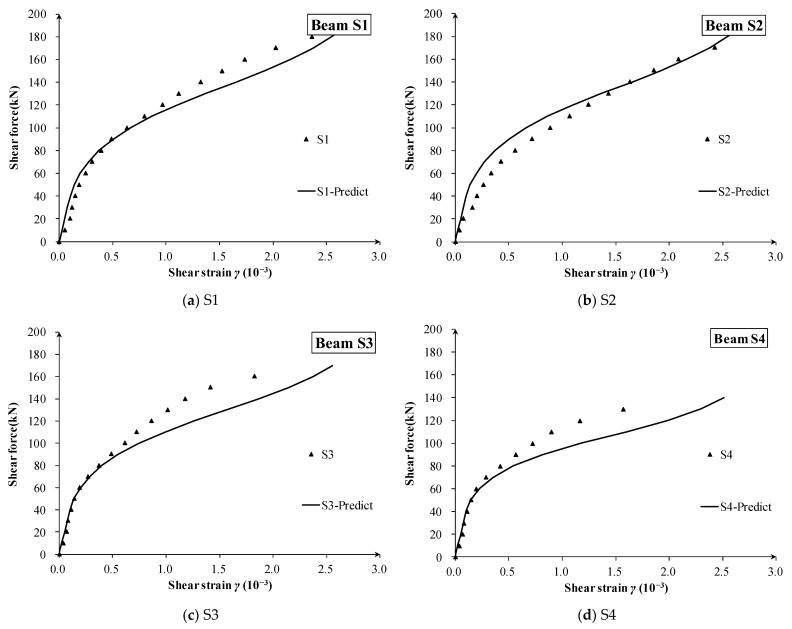
The shear strain of each measuring lattice in specimens S1, S2, S3 and S4.

The measured and calculated shear strain of 16 zones of 10 beams are shown in Figure 9, Figure 10 and Figure 11, respectively. Compared with the measured shear force–strain, the theoretical prediction results are in good agreement with the measured values. It can be concluded that the proposed shear stiffness degradation model simulates the degradation process of shear stiffness very well, and it tends to give a conservative prediction for the shear strain after diagonal cracking.

For the specimen CV series (Figure 9) and S series (Figure 11) with constant depth, the proposed degradation model is a little conservative and gives very acceptable accuracy. Meanwhile, the degradation rule well captures the main characteristics of the shear strain curves, such as the turning point for the first diagonal crack and the gradual evolution from elastic stiffness to fully diagonally cracked stiffness.

For the specimen BV series with variable depth (Figure 10), it should be noted that the inclined lower chord bears part of the shear force, which is not completely consistent with the assumption for constant depth beam, for which the concrete web bears most of the shear force based on elastic beam theory. Therefore, it can be foreseen that the shear deformation prediction of the BV series specimens will be slightly larger than experimental results in the early loading stage, but the degradation law of shear stiffness is still in good agreement among each specimen.

In addition, although the shear span ratios of different observed beam lattices have large changes (such as lattices G3 and G4 of BV series in Figure 9, and lattices S1 and S2 in Figure 10), there is no obvious relationship between measured shear strain and shear depth-to-span ratio. This shows that the shear depth-to-span ratio has little effect on the degradation of the shear stiffness, and its effect on the after-cracking shear deformation is less.

As the beams crack randomly during the loading stage, they cannot get into a fully diagonally cracked status that matches the theoretical assumptions. The real shear stiffness of the beams might be larger than theoretical values, while the real shear deformations are just the opposite. In total, the proposed model will give a relatively conservative shear stiffness prediction for partially diagonal cracked beams.

**Figure 12:**
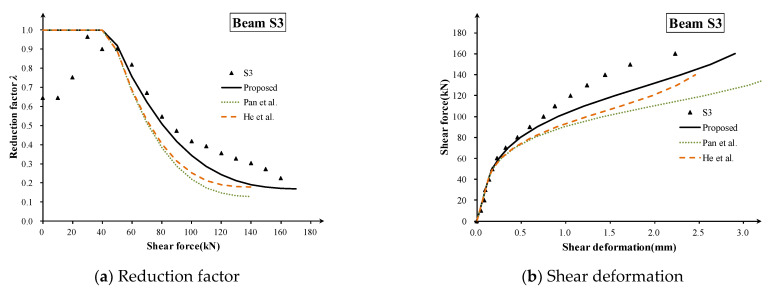
Comparison of three methods in prediction of shear stiffness and shear strain [8,9].

Here, specimen S3 (*ρ_s_* = 2.13%) is taken as an example to show the validation process. The calculated *V_cr_* = 45kN, and the calculated *V_y_* for the proposed method, Pan method and He method are 179.4 kN, 148.7 kN and 144.3 kN, respectively, while the values of *λ_u_* are 0.169, 0.127 and 0.178, respectively. The predicted ultimate shear stiffness degradation reduction factor *λ_u_* and shear strain calculated by three methods are compared to the measured data (Figure 12), which shows that the proposed method is a little better than others for the after-cracking shear stiffness evaluation.

In summary, the shear stiffness degradation model proposed in this paper can better evaluate the shear stiffness degradation of each beam lattice, and it can give a reasonable prediction of shear deformation, which can be used for the evaluation of the shear stiffness of diagonally cracked RC beams.

## 5. Conclusions

This study aims to obtain the shear stiffness degradation law and to propose a simplified and practical prediction method of thin-webbed beams, which can be further applied to existing concrete box-girder bridges with diagonal web cracks. Six large-scale thin-webbed concrete beams were tested in this paper to investigate the shear stiffness degradation of RC beams before and after shear cracking. Considering the effect of concrete tension stiffening, a practical shear stiffness degradation model was proposed and validated. The main conclusions can be drawn as follows:

1. The shear deformation test showed that the shear stiffness drops to about 30~40% of the original stiffness following the occurrence of the first main diagonal crack, and it further drops to only about 10% of the original stiffness when the stirrup yields.
2. The strut angle *θ_u_* was deduced by combining CFT and elastic beam theory. Compared with two other methods from the literature, the proposed angle tends to give a moderate prediction of strut angles and shear deformation with higher accuracy.
3. Considering the tensioning stiffness effect, a simplified shear stiffness degradation rule was suggested for a diagonally cracked RC beam. A cubic form degradation equation consistent with the degradation form of flexural stiffness was established and validated.
4. Data for a total of 16 zones of lattice shear deformation from 10 beams were measured or collected for verification. The results showed that a turning point occurs in the shear deformation curve corresponding to the first diagonal crack. And, rather than the pre-cracking stage, the shear span-to-depth ratio has little effect on the shear deformation of RC beams in the post-cracking stage.
5. The results showed that the proposed method gives a good and consistent prediction of the effective shear stiffness and shear strain development. The proposed model could capture the development characteristics of shear deformation curves. However, for the BV series, the bottom flanges bear part of the shear force, which will cause a larger predicted shear strain.
6. In general, the proposed simplified shear degradation model tends to give a conservative prediction of shear stiffness, and it is very practical for the early evaluation of diagonally cracked box-girder bridges in service.

Moreover, the contribution of the inclined flanges of variable depth specimens on shear capacity and shear deformation could be an interesting topic for further study. Also, the digital image correlation (DIC) technique maybe a good choice for shear deformation tests in future studies [38,39,40,41].

## Figures and Tables

**Table 1:** Details of specimens.

Resources	Specimen NO.	*f’_c_* (MPa)	*E_c_* (GPa)	*d_v_* (mm)	*b_w_* (mm)	*f_yv_* (MPa)	*ρ_v_* (%)	*ρ_s_* (%)	*M*/(*Vd*)	*θ_u_* (Degree)	*λ_u_*
Author	BC1-G3	39.0	29.4	684	100	327	0.5	4.8	0.4	26.3	0.182
	BC1-G4	39.0	29.4	684	100	327	0.5	4.8	0.4	26.3	0.182
	BC2-G3	36.0	28.2	684	100	327	0.4	4.8	0.4	25.2	0.169
	BC2-G4	36.0	28.2	684	100	327	0.4	4.8	0.4	25.2	0.169
	BV1-G3	39.0	29.4	543.6	100	327	0.5	6.0	1.2	25.8	0.184
	BV1-G4	39.0	29.4	450.9	100	327	0.5	7.23	0.4	25.4	0.185
	BV2-G3	39.0	29.4	543.6	100	327	0.4	6.0	1.2	24.6	0.168
	BV2-G4	39.0	29.4	450.9	100	327	0.4	7.2	0.4	24.3	0.169
	BV3-G3	36.0	28.2	543.6	100	327	0.5	6.0	1.2	25.9	0.187
	BV3-G4	36.0	28.2	450.9	100	327	0.5	7.2	0.4	25.6	0.188
	BV4-G3	36.0	28.2	543.6	100	327	0.4	6.0	1.2	24.7	0.171
	BV4-G4	36.0	28.2	450.9	100	327	0.4	7.2	0.4	24.4	0.172
Hansapinyo et al. [24]	S1	33.0	27.0	320	150	370	0.47	4.26	2.6	26.4	0.179
	S2	33.0	27.0	320	150	370	0.47	4.26	3.5	26.4	0.179
	S3	33.0	27.0	320	150	370	0.47	2.13	2.6	28.5	0.170
	S4	33.0	27.0	320	150	370	0.31	2.13	2.6	26.1	0.142

## Data Availability

Data will be requested to the authors.
